# Germination screening of nine annual Amaranthaceae halophytes identifies candidates for saline-alkali tolerant crop development

**DOI:** 10.3389/fpls.2026.1755439

**Published:** 2026-04-21

**Authors:** Xinyue Zhou, Hang Xiang, Xiaojun Yu

**Affiliations:** 1 Pratacultural College, Gansu Agricultural University, Lanzhou, China; 2 Academy of Water Resources Conservation Forests in Qilian Mountains of Gansu Province, Zhangye, China

**Keywords:** combined salt-alkali stress, comprehensive evaluation, halophyte, screening, seed germination

## Abstract

**Introduction:**

Screening of halophytes is the key to biological improvement of saline-alkali soils. This study sought to determine the influence of low, moderate, and high combined saline-alkaline stress on the germination period of different halophytes and evaluate the salt-alkali tolerance of halophytes.

**Methods:**

The paper-based culture method be used to determine the germination and growth indices of 9 halophytes under low, moderate, and high combined saline-alkaline stress.

**Results:**

The study emphasizes (1) the germination strategies of the 9 annuals halophytes showed significant differentiation, falling into 2 categories: “rapid-type” and “slow-type”. Seeds of *Suaeda corniculata*, *Halogeton arachnoideus* (LZ), and *Chenopodium quinoa* belonged to “rapid-type”, completing germination within merely 1~3 days. (2) The combined saline-alkali stress exerted a typical “low-promotion and high-inhibition” effect. Under moderate stress (MS, salt content 0.55%, pH 8.90, EC 6.38 mS cm^-1^), the fresh weight of *Salicornia europaea*, *Atriplex centralasiatica* and *Chenopodium glaucum* was significantly higher than that of low stress (LS, salt content 0.27%, pH 8.03, EC 3.62 mS cm^-1^) and high stress (HS, salt content 0.73%, pH 9.63, EC 9.54 mS cm^-1^) treatments. Specifically, compared with the LS treatment, the fresh weights were increased by 18.73%, 9.87%, and 13.42% for the three species, respectively; compared with the HS treatment, the corresponding increases were 71.76%, 88.95%, and 127.46%, respectively. High stress preferentially inhibited radicle length, thereby affecting biomass. Taking *Atriplex centralasiatica* and *Chenopodium quinoa* as examples, the inhibition rate of radicle length (94.19%, 95.57%) was significantly higher than that of inhibition rate of germ length (40.54%, 32.48%). (3) Correlation analysis revealed a positive correlation between seed germination vigor and root development along with biomass accumulation. Seed germination performance in saline-alkali environments is determined by the plant’s genetic background, the external stress intensity, and their complex interaction.

**Conclusions:**

*Suaeda salsa* demonstrated the strongest overall tolerance, while *Suaeda corniculata*, *Salicornia europaea*, and *Halogeton arachnoideus* are recommended for rehabilitating high, moderate, and low saline-alkali soils, respectively. This study elucidates halophyte germination strategies under combined saline-alkali stress, providing a theoretical basis toward effective species selection for saline-alkali land bioremediation.

## 1 Introduction

Saline-alkali land represents a vast potential reserve of cultivable land and a highly valuable “future grain source” for China (Han et al., 2025; Lei et al., 2025). Soil salinization poses a bottleneck problem, restricting the efficient utilization of land resources and the high-quality development of agriculture (Cuevas et al., 2019). In China, the composition of saline-alkali soils is extremely complex (Wang et al., 2011). with salinization and alkalization often occurring simultaneously. Consequently, most saline-alkali lands here are classified as compound types (Guo et al., 2025). In the arid northwest region of China, the primary ions responsible for salt damage are Cl^-^, Na^+^, and SO_4_²^-^, while HCO_3_^-^ and CO_3_²^-^ are the main ions causing alkali damage (Zhang et al., 2018). After years of reclamation efforts, mildly saline-alkali lands have largely been converted into productive farmland. However, moderately and severely saline-alkali lands remain underutilized due to persistently high soil salinity, deep mineralization of groundwater, and limited crop diversity (Petmuenwai et al., 2024; Reigosa et al., 2019). Although various halophytic plants have demonstrated potential for ameliorating saline-alkali soils (Rahman et al., 2021), research has predominantly focused on controlled environments subject to single-salt stress (notably NaCl) (Sobhanian et al., 2010; Wang et al., 2024; Wu et al., 2020) or on specific saline-alkali soils in particular regions (Díaz et al., 2013; Liang, 2023; Zhou, 2024). Consequently, there is insufficient screening of halophytes on different degrees of saline-alkali lands, which restricts the improvement and utilization of saline soils.

Halophytic plants are defined as those capable of surviving and completing their life cycle in high salt concentrations (200 mM NaCl) (Van Zelm et al., 2020), having developed various adaptive mechanisms that integrate physiological, biochemical, and morphological traits to cope with salt stress (Sharma et al., 2024). Annual halophytes, owing to their short life cycle, experimental tractability, reproductive efficiency, and genetic transformability, serve as key model organisms for salt tolerance research (Yuan et al., 2019). Currently, halophytes used for saline-alkali land remediation primarily fall into two categories: eu-halophytes and pseudo-halophytes (Luo et al., 2021; Wang et al., 2024, Wang et al., 2023b). The study of Hu et al. (2024) demonstrated that eu-halophytes are optimal for saline-alkali land restoration, outperforming pseudo-halophytes. For instance, *Suaeda salsa* accumulated the highest Na^+^ content in shoots and roots, while its soil Na^+^ uptake capacity, was significantly higher than that of *Reaumuria songarica*.

The most critical stage of planting halophytes to a saline-alkali land is germination and whether seeds can healthily, orderly germinate (Qi et al., 2023). A previous study has demonstrated that the seed germination period is the most sensitive and susceptible to environmental factors in the entire growth period of plants (Baudouin et al., 2022). Therefore, it is of great significance to undergo saline-alkali complex stress during the seed germination period (Nisar et al., 2021). The tolerance limits for salinity in different halophytic plants are different during the germination period, adapting to saline stress environments by regulating osmosis through the accumulation of high concentrations of Na^+^, and the characteristics of the same plants under different salinity are also different. For *Suaeda salsa*, the optimal growth of 200 mM NaCl is required, which can accumulate concentrations of Na^+^ to 400 mM NaCl without being damaged (Song and Wang, 2015). The study of Sagers et al. (2017) posited that the *Halogeton arachnoideus*’ aboveground biomass increased at a salt concentration of 400 mM NaCl, osmoregulation was performed by accumulation of Na^+^ and tolerated salt stress up to 800 mM NaCl. The germination of salinity plants such as *Chenopodium quinoa* (Brakez et al., 2014)*, Halogeton arachnoideus* (Zhao et al., 2021) wait in response to salinity stress is mainly concentrated on single salt treatment or two mixed salt treatment or single salinity plants on salinity and alkali complex stress.

However, comparative analyses of the responses of eu-halophytes and pseudo-halophytes to varying saline-alkaline conditions remain limited, particularly during the critical seed germination stage. To address this gap, this study simulate a combined salt-alkali stress environment, this study created soil conditions representing low, moderate, and high saline-alkali levels (Wang, 2024; Xu et al., 2023) by combining two neutral salts (NaCl and Na_2_SO_4_) and two alkaline salts (Na_2_CO_3_ and NaHCO_3_) in different ratios. The aim is to investigate the emergence characteristics of eu-halophytes and pseudo-halophytes under different combined salt-alkali stress, and to screen the best-adapted plant species under different composite stress conditions, and the results of the study will provide theoretical support for the improvement of bioremediation of saline and alkaline soils with different intensities.

## 2 Materials and methods

### 2.1 Experimental materials

A total of 10 seed samples, representing 9 halophytic plant species, were used in this study. The species were categorized as follows: eu-halophytes: *Suaeda salsa* (black seed), *Suaeda corniculata* (black seed), *Salicornia europaea*, and *Halogeton arachnoideus*; pseudo-halophytes: *Atriplex centralasiatica* (flat-black seed), *Chenopodium glaucum*, *Chenopodium rubrum*, *Grubovia dasyphylla*, *Chenopodium quinoa* (**Table 1**). All were collected at the seed maturation stage and are stored in a seed storage box with a temperature of 5 °C and a humidity of 27%.

**Table 1:** List of tested halophyte species and their origins.

Number	Latin name	Genus	1000 grain weight (g)	Longitude	Latitude	Altitude (m)
1	*Suaeda salsa*	*Suaeda*	0.17	103°15′50″ E	38°28′53″ N	1325.61
2	*Suaeda corniculata*	*Suaeda*	1.93	103°15′50″ E	38°28′54″ N	1313.92
3	*Salicornia europaea*	*Salicornia*	0.40	103°15′50″ E	38°30′27″ N	1325.61
4	*Halogeton arachnoideus*	*Halogeton*	0.43	103°46′03″ E	36°28′14″ N	1948.46
5	*Halogeton arachnoideus*	*Halogeton*	0.29	104°15′48″ E	37°15′48″ N	1510.81
6	*Atriplex centralasiatica*	*Atriplex*	8.04	103°53′13″ E	38°17′29″ N	1317.21
7	*Chenopodium glaucum*	*Chenopodium*	0.55	103°15′50″ E	38°28′54″ N	1313.92
8	*Chenopodium rubrum*	*Chenopodium*	0.69	103°02′14″ E	38°35′10″ N	1322.18
9	*Grubovia dasyphylla*	*Bassia*	1.02	102°53′14″ E	38°42′23″ N	1305.96
10	*Chenopodium quinoa*	*Chenopodium*	2.66	–	–	–

Seeds of *Halogeton arachnoideus* were represented by two distinct populations: *H. arachnoideus* (LZ) collected from Lanzhou City (36°28′14″ N, 103°46′03″ E, salt content 0.38%, pH 8.42) and *H. arachnoideus* (WW) from Wuwei City (37°15′48″ N, 104°15′48″ E, salt content 0.34%, pH 7.94) in Gansu Province in 2024. Seeds of the remaining 7 species were collected in Gansu Province in 2023. The seeds of *Chenopodium quinoa* were provided by the Gansu Academy of Agricultural Sciences.

### 2.2 Experimental design

Combining the classification standards of the United States Labs (USSL) (based on Electrical Conductivity values) (Bashir et al., 2020; Vargas et al., 2018) with the Chinese classification standards for saline-alkali soils (based on salt content) (Wang, 2000; Yang et al., 2022). This experiment was controlled by the addition of no saline as a control (CK), and low combined salt-alkali stress was set: LS (NaCl: Na_2_SO_4_:NaHCO_3_:Na_2_CO_3_ = 1:2:1:0, 25 mM, salt content 0.27%, pH 8.03, EC = 3.62 mS cm^-1^), moderate combined salt-alkali stress: MS (NaCl: Na_2_SO_4_:NaHCO_3_:Na_2_CO_3_ = 1:9:9:1, 50 mM, salt content 0.55%, pH 8.90, EC = 6.38 mS cm^-1^), high combined salt-alkali stress: HS (NaCl: Na_2_SO_4_:NaHCO_3_:Na_2_CO_3_ = 1:1:1:1, 75 mM, salt content 0.73%, pH 9.63, EC = 9.54 mS cm^-1^).The pH values and EC values of each treatment were measured separately using a pH meter and a conductivity meter (model DZS-706F-A). The experiment employed the paper-based culture method. Mature, plump, and healthy seeds were selected and surface-sterilized with 75% ethanol for 30 s, followed by rinsing five times with distilled water. The seeds were then air-dried at room temperature on filter paper. Sterile Petri dishes with a diameter of 90 mm were used, with a layer of absorbent cotton placed at the bottom as a water-absorbing layer, topped with two pieces of filter paper. 20 mL of treatment solution were added to each Petri dish. Fifty uniformly sized seeds were selected and evenly distributed in each dish, with three replicates prepared. The initial weight of each Petri dish was measured. The test Petri dishes was placed in an incubator (model HGZ-HS250) at Gansu Agricultural University (Gansu, China) in May 2025. With germination test in an incubator under a photoperiod of 12 h light (25°C) and 12 h dark (20°C), 65% relative humidity, and a light intensity of 6000 Lx. According to the weighing water method (Li et al., 2023; Liu et al., 2026; Martínez-Ballesta et al., 2020), the Petri dish was weight every 24 h, and lost water was supplemented to maintain the constant solution concentration in the Petri dish. The seed germination and seedling growth were observed and recorded. The cumulative germination rate of the previous 3 days reached a plateau was classified as “fast-track”, and conversely, it was classified as “slow-track”.

### 2.3 Data processing and evaluation methods of indicators

During seed germination, the emergence of germ length exceeding half its own length is used as an indicator of seed germination. Germination counts for each treatment are recorded daily at 6:00 PM. Germination potential (GP) (Equation 1) is calculated on the 4^th^ day, and germination rate (GR) (Equation 2) is calculated on the 7^th^ day. Measurements include radicle length (RL), germ length (GL), fresh weight (FW), dry weight (DW), germination index (GI) (Equation 3), vigor index (VI) (Equation 4), and relative salinity damage rate (RSDR) (Equation 5) (Gao et al., 2025; Khan et al., 2022).

(1) $$\begin{array}{c} GP(\%)=\frac{The\;normal\;number\;of\;germinated\;seeds}{The\;total\;number\;of\;seeds\;tested}\times 100 \end{array}$$

(2) $$\begin{array}{c} GR(\%)=\frac{The\;normal\;number\;of\;germniated\;seeds\;that\;reached\;the\;peak}{The\;total\;number\;of\;seeds\;tested}\times 100 \end{array}$$

Randomly select 10 healthy seedlings from each petri dish, gently remove the seed coat covering the seedlings, measure the radicle length, and germ length using a ruler to the nearest 0.01 mm, weigh the fresh weight using a precision balance with an accuracy of 0.01 mg, place them in an envelope, and subject them to heat treatment at 105 °C for 30 min, followed by drying at 65 °C until constant weight is achieved, and then measure the dry weight.

(3) $$\begin{array}{c} GI=\sum \frac{G_{t}}{D_{t}} \end{array}$$

(4) $$\begin{array}{c} VI(cm)=S\times GI \end{array}$$

Where *Gt* is defined as the number of seeds germinated on day t.; *Dt* is the days to germination; *S* is the length of seedlings, *S=RL+GL*.

(5) $$\begin{array}{c} RSDR(\%)=\frac{\left(germination\;rate\;of\;controlled\;-\;germination\;rate\;of\;stressed\right)}{germination\;rate\;of\;controlled}\times 100 \end{array}$$

Based on the values of GR, GP, GI, VI, RL, GL, FW, and DW, the following indices were calculated: relative germination rate (RGR), relative germination potential (RGP), relative germination index (RGI), and relative vigor index (RVI); relative germ length (RGL), relative radicle length (RRL), relative fresh weight (RFW), and relative dry weight (RDW). The relative value for each indicator was calculated as the ratio of the value in the treatment group to that in the control group (Lei et al., 2025).

Statistical analysis was performed using SPSS 27.0 software (IBM SPSS Statistics, IBM corp., Chicago, IL, USA), with Duncan’s multiple range test (*p*<0.05) and two-way analysis of variance (ANOVA) to assess the interaction effects between factors. Figures and tables were created using Origin 2024 (Origin Lab, Northampton, MA, USA) and Microsoft Excel 2021 (Microsoft Co., Ltd., Redmond, WA, USA). Following the method described by Chen et al. (2023), the membership function values, weight coefficient, and comprehensive evaluation value for each plant’s comprehensive index were calculated using equations (6) ~ (10), respectively. The calculation formulas are as follows:

RGR, RGP, RGI, RVI, RGL, RRL, RFW, and RDW are positively correlated with resistance and are calculated using Equation 6, while RSDR is negatively correlated with resistance and is calculated using Equation 7.

(6) $$\begin{array}{c} U(X_{i})\;=\;\frac{X_{i}\;-X_{min}}{X_{max}\;-\;X_{min}} \end{array}$$

(7) $$\begin{array}{c} U(X_{i})\;=\;1\;-\;\frac{X_{i}\;-\;X_{min}}{X_{max}\;-\;X_{min}} \end{array}$$

Where *U(X_i_)* is the membership function value of a certain measurement index; *X_i_* is the measured value of that index; *X_max_* is the maximum value of that index; *X_min_* is the minimum value of that index.

Contribution rate (Equation 8):

(8) $$\begin{array}{c} V_{i}\;(\%)=\frac{S_{i}}{{\bar{X}}_{i}}\times 100 \end{array}$$

Weight coefficient (Equation 9):

(9) $$\begin{array}{c} W_{i}=\frac{V_{i}}{\sum_{i=1}^{n}V_{i}} \end{array}$$

Where *V_i_* is the contribution rate of the *i* th composite indicator, *S_i_* is the standard deviation of the *i* th indicator, and *W_i_* is the weight of the *i* th indicator.

Comprehensive evaluation value (Equation 10):

(10) $$\begin{array}{c} D\;=\;\sum_{i\;=\;1}^{n} \end{array}[U(X_{i})\;\;W_{i}]$$

Where *D* is the comprehensive evaluation value.

## 3 Results

### 3.1 Effect of combined salt-alkali stress on the germination dynamics of halophytes

**Figure 1:**
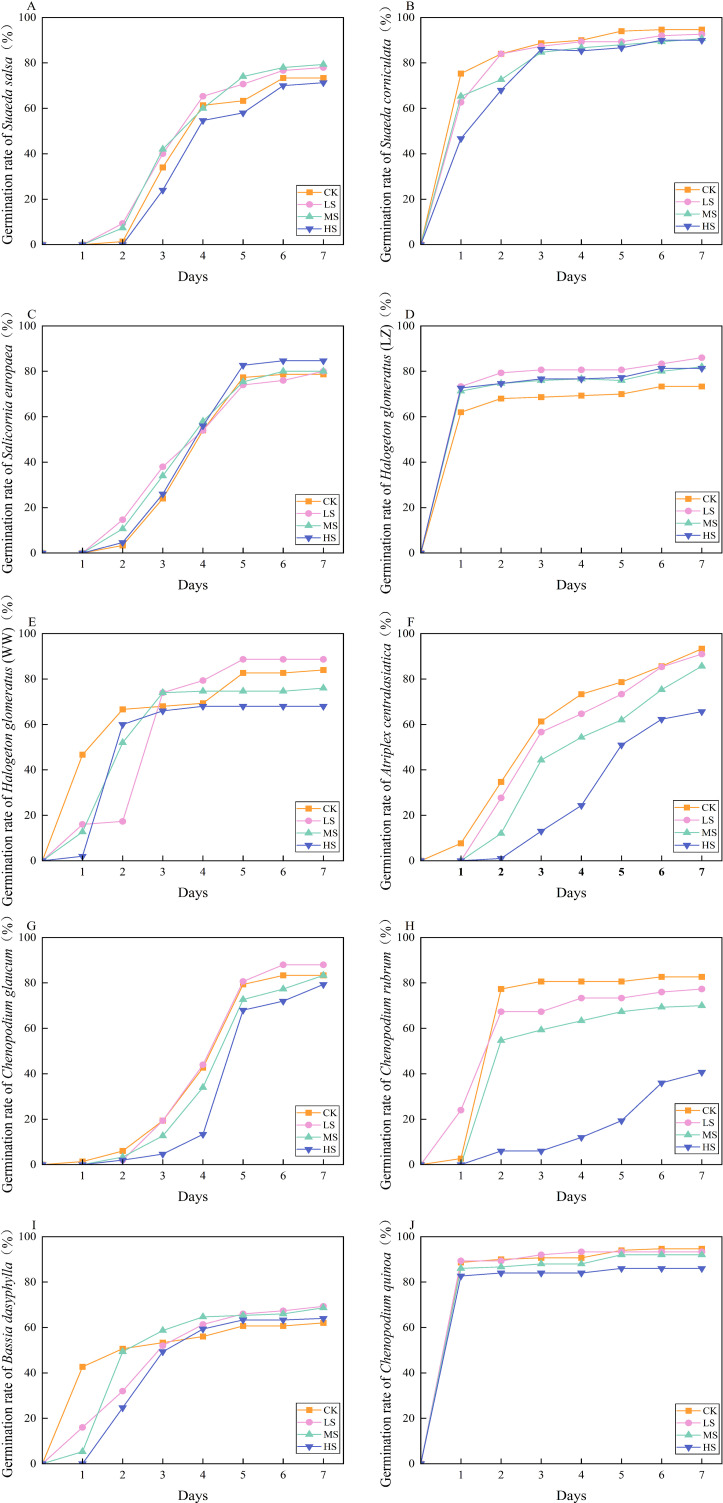
Germination dynamics of halophytes across a gradient of combined saline-alkaline stress. CK, control; LS, low combined salt-alkali stress; MS, moderate combined salt-alkali stress; HS, high combined salt-alkali stress.

Combined salt-alkali stress affects both the cumulative germination rate and germination speed of halophytic plant seeds (**Figure 1**). HS treatment prolonged the initial germination time of *S. salsa, A. centralasiatica*, and *G. dasyphylla. S. corniculata, H. arachnoideus* (LZ), and *C. quinoa* began germinating on the first day under all treatments; *S. salsa, S. europaea*, and *C. glaucum* began germination on the second day under all treatments; *H. arachnoideus* (WW), *A. centralasiatica*, and *G. dasyphylla* began germination on the first day under CK, LS, and MS treatments, and on the second day under HS treatment. *C. rubrum* began to germinate on the first day under LS treatment and on the second day under CK, MS, and HS treatments. The cumulative germination rates of *H. arachnoideus* (LZ), *H. arachnoideus* (WW), and *C. glaucum* reached their maximum values under LS treatment; the cumulative germination rates of *S. salsa* and *G. dasyphylla* reached their maximum values under MS treatment; and the cumulative germination rate of *S. europaea* reached its maximum value under HS treatment. The germination rates of *S. salsa*, *S. europaea*, *H. arachnoideus* (WW), *A. centralasiatica*, *C. rubrum*, *C. glaucum*, and *G. dasyphylla* increased slowly during the early stages of germination (3~5 days), while the germination rates of *S. corniculata*, *H. arachnoideus* (LZ), and *C. quinoa* seeds increased rapidly during the early stages of germination (1~3 days).

### 3.2 Effects of combined salt-alkali stress on germination indicators of halophytes

#### 3.2.1 Effects of combined salt-alkali stress on the germination potential of halophytes

**Figure 2:**
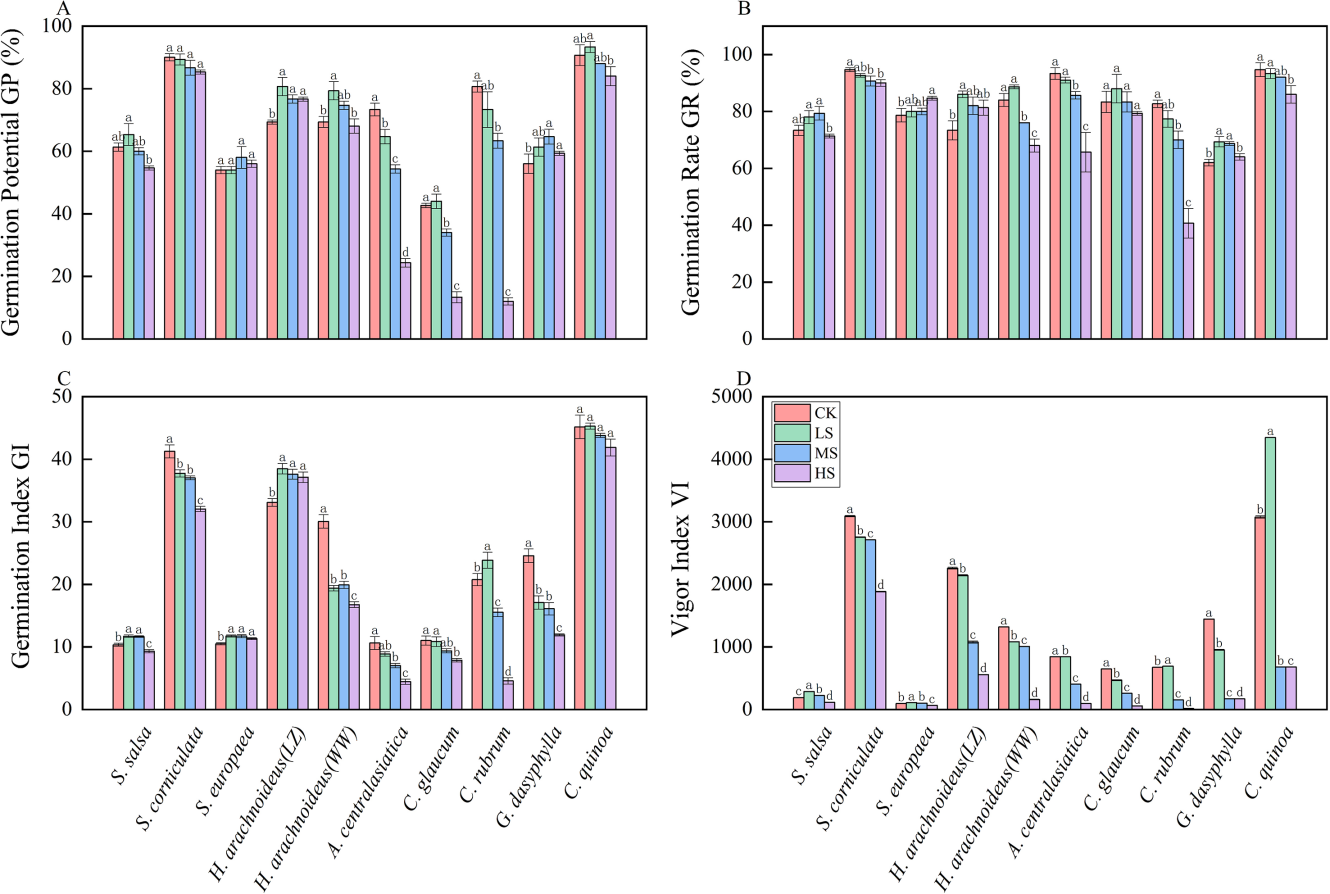
Comparative analysis of germination indicators of different halophytes under different combined saline-alkaline stress. **(A)** Germination Potential GP, **(B)** Germination Rate GR, **(C)** Germination Index GI, **(D)** Vigor Index VI. Statistical test used, letters indicate significant differences in diversity (*p*<0.05). CK, control; LS, low combined salt-alkali stress; MS, moderate combined salt-alkali stress; HS, high combined salt-alkali stress.

The germination potential of halophytes exhibited significant differences under combined salt-alkali stress (**Figure 2A**). Under all treatments, *S. corniculata* and *C. quinoa* maintained high germination potential (84.00~93.33), significantly higher than other halophytes (*p*<0.05). There were no significant differences in germination potential among the various combined salt-alkali stress treatments for *S. corniculata* (*p*>0.05). The germination potential of *S. salsa* and *H. arachnoideus* (WW) was higher under LS and MS treatments but significantly decreased under HS treatment (*p*<0.05). The germination potential of *A. centralasiatica, C. glaucum*, and *C. rubrum* showed a sharp decrease in germination potential under the HS treatment, significantly lower than under the LS and MS treatments (*p*<0.05), with reductions of 165.80%, 230.08%, and 511.08% compared to the LS treatment, and 120.30%, 155.06%, and 427.75% compared to the MS treatment. The germination potential of *H. arachnoideus* (LZ), *S. europaea*, and *G. dasyphylla* remained relatively stable under various saline-alkali composite stress treatments, with no significant differences (*p*>0.05).

#### 3.2.2 Effects of combined salt-alkali stress on the germination rate of halophytes

The germination rates of halophytes exhibited significant differences under combined salt-alkali stress (**Figure 2B**). Under all treatments, *S. corniculata* and *C. quinoa* maintained high germination rates (86.00% ~ 94.67%), which were significantly higher than other halophytes (*p*<0.05). The germination rate of *S. europaea* was highest under the HS treatment (84.67%), and was 80% under both LS and MS treatments, with no significant difference compared to the HS treatment (*p*>0.05). The germination rates of *S. salsa* and *G. dasyphylla* were higher under LS and MS treatments, significantly higher than under the HS treatment (*p*<0.05). The germination rates of *H. arachnoideus* (LZ) and *C. glaucum* gradually decreased with increasing combined salt-alkali stress intensity, but the differences were not significant (*p*>0.05). The germination rates of *H. arachnoideus* (WW), *A. centralasiatica*, and *C. rubrum* were highest under CK and LS treatments, and showed a significant decreasing trend with increasing combined salt-alkali stress intensity (*p*<0.05).

#### 3.2.3 Effects of combined salt-alkali stress on the germination index of halophytes

The germination index of halophytes exhibited significant differences under combined salt-alkali stress (**Figure 2C**). Under all treatments, *C. quinoa* maintained a high germination index, with no significant differences observed among treatments (*p*<0.05). The germination index of *S. europaea* remained relatively stable under all combined salt-alkali stress treatments and was higher than that of the CK. The germination index of *C. glaucum* and *S. salsa* were higher under LS and MS treatments, with no significant differences (*p*>0.05), and gradually decreased as the intensity of combined salt-alkali stress increased. The germination index of *C. rubrum* was highest under the LS treatment. The germination index of *H. arachnoideus* (WW) and *A. centralasiatica* were significantly lower than the CK under all combined salt-alkali stress treatments (*p*<0.05), and gradually decreased with increasing intensity of combined salt-alkali stress.

#### 3.2.4 Effects of combined salt-alkali stress on the vigor index of halophytes

The vigor index of halophytes exhibits significant differences under combined salt-alkali stress (**Figure 2D**). As the intensity of combined salt-alkali stress increased, the vigor index of most halophytes showed a decreasing trend, but the extent of decrease varied significantly among different plants (*p*<0.05). The vigor index of *S. salsa*, *S. europaea*, *C. rubrum*, and *C. quinoa* were significantly higher under LS treatment than under other treatments (*p*<0.05), and they gradually decreased as the intensity of combined salt-alkali stress increased. The vigor index of *S. corniculata*, *H. arachnoideus* (LZ), *H. arachnoideus* (WW), *A. centralasiatica*, *C. glaucum*, and *G. dasyphylla* were significantly lower than those under the CK under all combined salt-alkali stress conditions (*p*<0.05), and the vigor index gradually decreased as the intensity of combined salt-alkali stress increased. *S. corniculata* maintained a high vitality value of 1,884.59 under the HS treatment, representing a 39.0% decrease compared to the CK. The vigor index of *C. rubrum* and *G. dasyphylla* plummeted to 15.11 and 170.56, respectively, under the HS treatment.

### 3.3 Effects of combined salt-alkali stress on the growth indicators of halophytes

#### 3.3.1 Effects of combined salt-alkali stress on the germ length of halophytes

**Figure 3:**
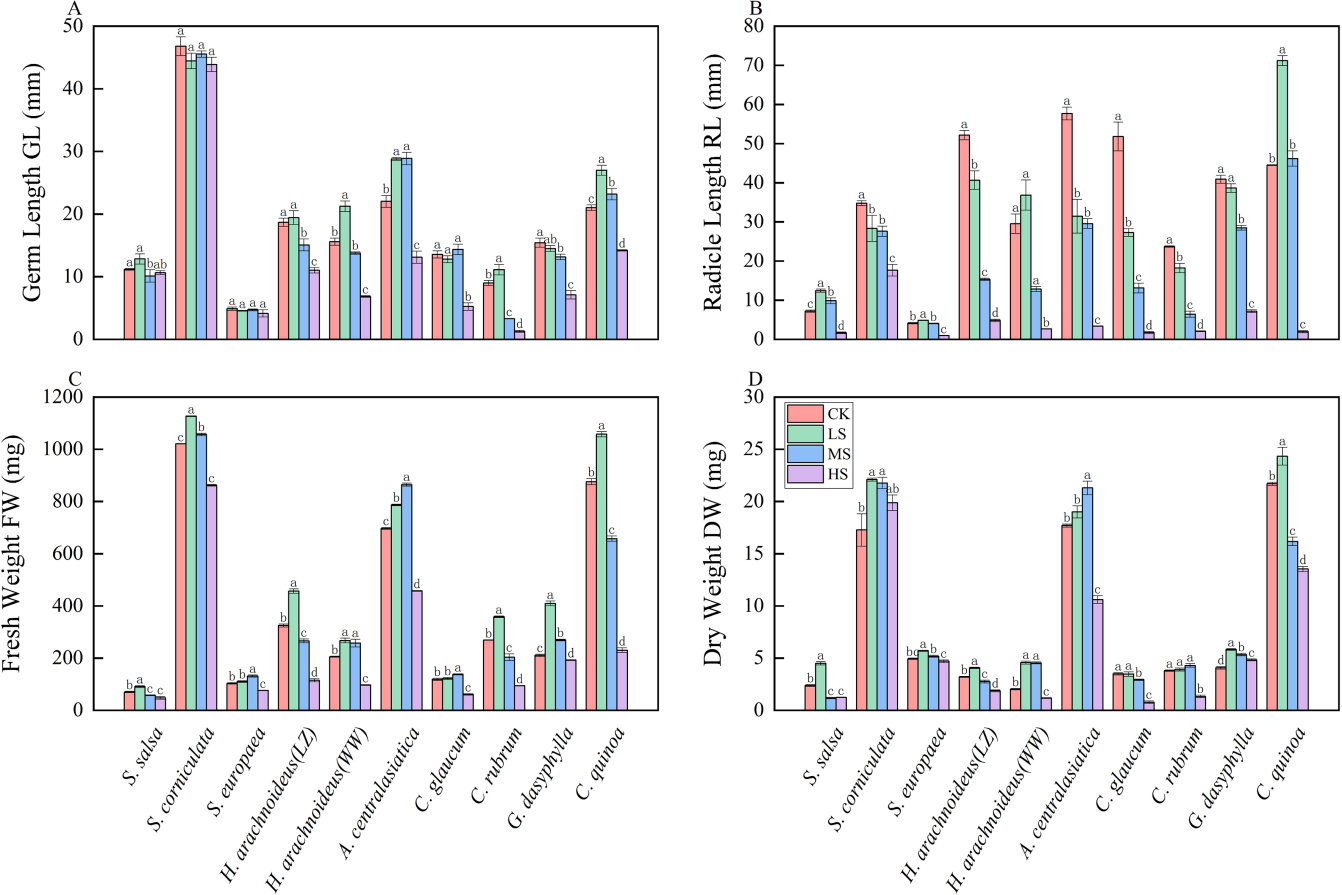
Comparative analysis of growth indicators of different halophytes under different combined saline-alkaline stress. **(A)** Germ Length GL, **(B)** Radicle Length RL, **(C)** Fresh Weight FW, **(D)** Dry Weight DW. Statistical test used, letters indicate significant differences in diversity (*p*<0.05). CK, control; LS, low combined salt-alkali stress; MS, moderate combined salt-alkali stress; HS, high combined salt-alkali stress.

**Figure 4:**
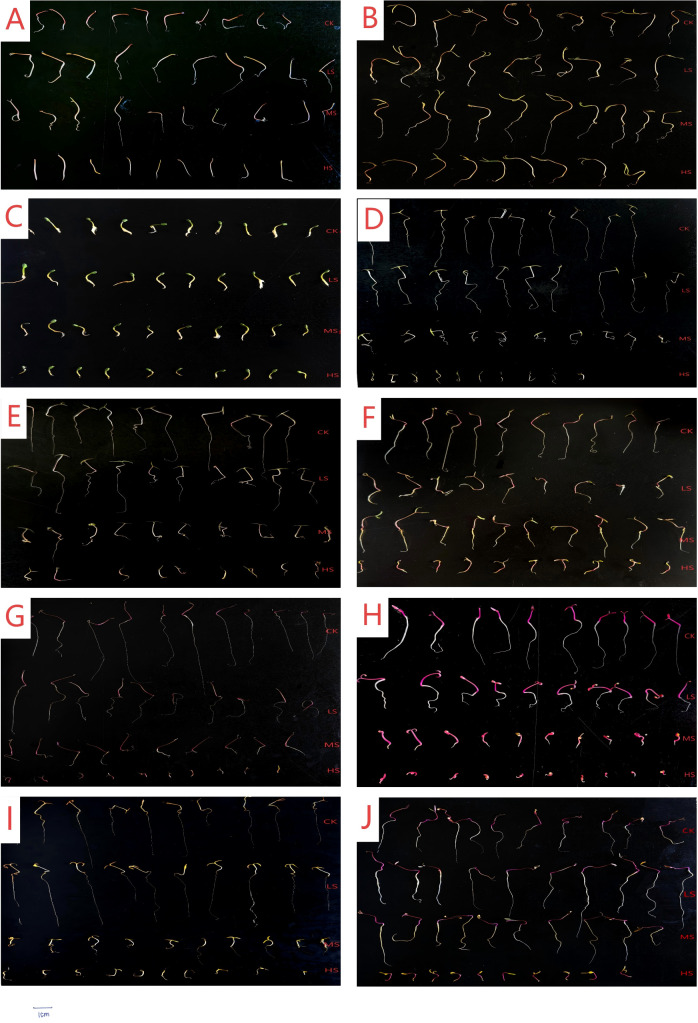
Growth status of halophyte seedlings at the germination stage under combined saline-alkaline stress. **(A)** *Suaeda salsa*; **(B)** *Suaeda corniculata*; **(C)** *Salicornia europaea*; **(D)** *Halogeton arachnoideus* (LZ); **(E)** *Halogeton arachnoideus* (WW); **(F)** *Atriplex centralasiatica*; **(G)** *Chenopodium glaucum*; **(H)** *Chenopodium rubrum*; **(I)** *Grubovia dasyphylla*; **(J)** *Chenopodium quinoa*. CK, control; LS, low combined salt-alkali stress; MS, moderate combined salt-alkali stress; HS, high combined salt-alkali stress.

The germ length of halophytes under combined salt-alkali stress showed significant differences (**Figures 3A**, **4**). As the intensity of combined salt-alkali stress increased, the germ length of most halophytes decreased, but the extent of decrease varied significantly among different plant species (*p*<0.05). *S. salsa* exhibited the longest germ length under LS treatment, but the differences compared to CK and HS treatments were not significant (*p*<0.05). Under all combined salt-alkali stress, there were no significant differences in the germ lengths of *S. europaea* and *S. corniculate* (*p*>0.05), and *S. corniculata* was significantly higher than other halophytes (*p*<0.05), reaching 46.80 mm. The germ lengths of *H. arachnoideus* (LZ), *H. arachnoideus* (WW), *C. rubrum*, and *C. quinoa* were significantly higher under LS treatment than under other treatments (*p*<0.05). The germ lengths of *A. centralasiatica* and *C. glaucum* were longest under MS treatment, with no significant difference compared to LS treatment (*p*>0.05).

#### 3.3.2 Effects of combined salt-alkali stress on the radicle length of halophytes

The radicle length of halophytes under combined salt-alkali stress showed significant differences (**Figures 3B**, **4**). As the intensity of combined salt-alkali stress increased, the radicle length of most halophytes decreased, but the extent of decrease varied significantly among different plant species (*p*<0.05). The radicle length of *S. salsa*, *S. europaea*, and *C. quinoa* were significantly higher under LS treatment compared to CK, MS, and HS treatments (*p*<0.05). The radicle length of *S. corniculata*, *H. arachnoideus* (LZ), *A. centralasiatica*, *C. glaucum*, *C. rubrum*, and *G. dasyphylla* were significantly lower under saline-alkali stress compared to the CK (*p*<0.05), and both radicle length decreased gradually as the intensity of combined salt-alkali stress increased. The root lengths of *H. arachnoideus* (WW) and *C. quinoa* were higher than those of the CK under the LS treatment. The radicle length of *S. corniculata* reached 17.67 mm under the HS treatment, significantly higher than other plants (*p*<0.05), and 49.2% lower than CK.

#### 3.3.3 Effects of combined salt-alkali stress on the fresh weight of halophytes

The fresh weight of halophytes exhibited significant differences under combined salt-alkali stress (**Figure 3C**). As the intensity of combined salt-alkali stress increased, the fresh weight of most halophytes showed a decreasing trend; however, the extent of decrease varied significantly among different halophytes (*p*<0.05). The fresh weight of all halophytes under the LS treatment was higher than that under the CK. The fresh weight of *S. corniculata*, *C. quinoa*, *S. salsa*, *H. arachnoideus* (LZ), *H. arachnoideus* (WW), *C. rubrum*, and *G. dasyphylla* was highest under the LS treatment, significantly higher than under other treatments (*p*<0.05). with *S. corniculata* and *C. quinoa* reaching 1,126.60 mg and 1,057.87 mg, respectively. There was no significant difference in the fresh weight of *H. arachnoideus* (WW) between the LS and MS treatments (*p*>0.05). The fresh weight of S. europaea, *A. centralasiatica*, and *C. glaucum* was significantly higher under the MS treatment than under other treatments (*p*<0.05), and under the HS treatment, the fresh weight was lower than under the CK.

#### 3.3.4 Effects of combined salt-alkali stress on the dry weight of halophytes

The dry weight of halophytes exhibited significant differences under combined salt-alkali stress (**Figure 3D**). As the intensity of combined salt-alkali stress increased, the dry weight of most halophytes showed a decreasing trend; however, the extent of decrease varied significantly among different halophytes (*p*<0.05). *S. salsa*, *S. corniculata*, *S. europaea*, *H. arachnoideus* (LZ), *H. arachnoideus* (WW), *C. glaucum*, *G. dasyphylla*, and *C. quinoa* exhibited the highest dry weight under LS treatment; *C. quinoa* had the highest dry weight among all halophytes, at 24.33 mg; the dry weight of *S. corniculata* showed no significant differences under LS, MS, and HS treatments (*p*>0.05) and remained at a high level; the dry weight of *H. arachnoideus* (WW) significantly increased under LS and MS treatments compared to the CK (*p*<0.05). *A. centralasiatica* and *C. rubrum* had the highest dry weight under MS treatment, and *C. rubrum* showed no significant differences in dry weight under CK, LS, and MS treatments (*p*>0.05).

### 3.4 Effects of combined salt-alkali stress on relative indicators during seed germination

The relative indicators of halogen germination period differed significantly under combined salt-alkali stress (**Table 2**). The RGP, RGR, and RGI of *S. europaea* all reach 100 or above under LS treatment. *H. arachnoideus* (LZ) under LS treatment, RGP was 116.36% and RGR was 117.28%, while the germination indexes were significantly reduced under HS treatment, with significant inhibition effect (*p*<0.05). *C. glaucum* RGR was 100.00 under MS treatment, and significantly decreased under HS treatment (*p*<0.05). *S. salsa* reached 151.22% under LS treatment, and mild salinity was conducive to maintaining the vitality of the seedlings; *H. arachnoideus* (LZ) RVI was only 24.61% under HS treatment, and severe salinity seriously weakened the vitality. *S. europaea* RLL was 117.92% and RGL was 93.27% under LS treatment, and mild salinity promoted germ length and radicle length growth; *H. arachnoideus* (WW) RLL was 9.04% and RGL was 43.78% under HS treatment, and severe salinity was significantly inhibited. *S. salsa* under LS treatment was 129.44% and RDW was 189.87%, and mild salinity was conducive to substance accumulation; *C. rubrum* under HS treatment was 33.00%, and substance accumulation was significantly reduced under severe salinity. Most plants had RSDRs increased under HS treatment, *S. salsa* was 8.18% under HS treatment, *A. centralasiatica* was 29.64%, and *C. rubrum* reached 50.80%, indicating that severe salinity caused significant harm to plants; *C. glaucum* was 0.00% under MS treatment, while moderate salinity did not cause significant harm; *S. europaea*, *H. arachnoideus* (LZ), *G. dasyphylla* was treated with negative RSDRs under LS, MS, and HS treatment, and were - 7.63%, - 10.91%, and - 3.23% under HS treatment.

**Table 2:** Relative germination parameters of halophytes under low, moderate, and high combined saline-alkaline stress.

Halophyte	Treatment	RGP	RDR	RGI	RVI	RRL	RDL	RFW	RDW	RSDR
*S. salsa*	LS	106.52	106.37	113.24	151.22	172.64	114.86	129.44	189.87	-6.37
	MS	97.83	108.18	112.46	117.50	137.08	90.69	82.19	49.37	-8.18
	HS	89.14	97.27	89.95	60.29	23.19	95.17	67.94	51.90	2.73
*S. corniculata*	LS	99.26	97.89	91.49	89.15	81.41	94.94	110.38	127.97	2.11
	MS	96.30	95.77	89.74	87.70	79.31	97.29	103.52	126.06	4.23
	HS	94.81	95.07	77.67	60.98	50.78	93.74	84.42	115.06	4.93
*S. europaea*	LS	100.00	101.69	112.01	117.09	117.92	93.27	107.12	115.62	-1.69
	MS	107.00	101.69	111.82	108.35	97.58	95.92	127.18	104.87	-1.69
	HS	103.70	107.63	108.01	68.95	24.21	84.29	74.05	95.33	-7.63
*H. arachnoideus* (LZ)	LS	116.36	117.28	116.29	94.86	77.96	104.07	140.53	127.19	-17.28
	MS	110.59	111.82	113.69	47.66	29.33	80.72	81.74	85.94	-11.82
	HS	110.59	110.91	112.21	24.61	9.26	59.08	35.38	58.44	-10.91
*H. arachnoideus* (WW)	LS	114.42	105.56	64.49	82.08	124.75	136.22	130.34	225.12	-5.56
	MS	107.70	90.48	66.26	76.39	43.45	88.27	125.47	223.15	9.52
	HS	98.08	80.95	55.74	12.07	9.04	43.78	47.13	57.64	19.05
*A. centralasiatica*	LS	88.19	97.50	83.44	100.00	54.47	130.73	112.89	107.34	2.50
	MS	74.09	91.79	66.13	48.00	51.25	131.05	124.04	120.34	8.21
	HS	37.62	70.36	41.86	11.40	5.81	59.46	65.64	59.89	29.64
*C. glaucum*	LS	103.12	105.60	98.37	72.18	52.64	94.33	102.36	99.14	-5.60
	MS	79.68	100.00	85.03	39.87	25.35	105.90	116.09	83.71	0.00
	HS	31.24	95.20	71.42	8.95	3.34	38.54	51.04	22.00	4.80
*C. rubrum*	LS	90.90	93.54	114.89	102.85	76.82	123.67	133.29	102.63	6.46
	MS	78.51	84.67	74.84	22.63	27.10	36.67	75.76	113.16	15.33
	HS	14.88	49.20	22.02	2.25	8.72	14.11	35.03	35.00	50.80
*G. dasyphylla*	LS	109.52	111.82	69.53	65.91	94.45	93.97	194.60	143.24	-11.82
	MS	115.48	110.76	65.50	11.81	69.68	85.22	127.99	130.96	-10.76
	HS	105.95	103.23	48.54	11.81	17.60	46.01	91.34	118.67	-3.23
*C. quinoa*	LS	102.93	98.58	100.24	141.28	160.00	128.39	120.78	112.12	1.42
	MS	97.06	97.18	96.94	22.10	103.75	110.18	75.07	74.52	2.82
	HS	92.64	90.84	92.69	22.10	4.43	67.52	26.30	62.35	9.16

Different combined salt-alkali stress intensity had a significant effect on the germination and growth indicators of different plants (**Table 3**). The halophytes showed extremely significant differences in RGI, RVI, RFW and other indicators (*p*<0.01). The effects of combined salt-alkali stress intensity on RVI, RRL, RFW and other indicators also reached a very significant difference (*p*<0.01). The interaction between halophytes species and combined salt-alkali stress intensity had a very significant impact on germination and growth indicators (*p*<0.01), and the intensity of combined salt-alkali stress played a major role in seed germination and growth, the germination performance of seeds in saline-alkali environments is jointly determined by the plant’s genetic background, the intensity of external stress, and the complex interactions between the two factors.

**Table 3:** Two-way ANOVA (*F*-values) for the effects of combined saline-alkaline stress intensity and halophytes species on relative germination indices.

Factor	RDP	RGR	RGI	RVI	RRL	RGL	RFW	RDW	RSDR
Halophyte species	93.24**	38.22**	144.90**	1527.62**	88.82**	20.76**	101.23**	73.85**	38.22**
Stress intensity	155.33**	50.37**	176.90**	8816.96**	733.82**	172.57**	1431.61**	346.59**	50.37**
Halophyte species × Stress intensity	21.97**	6.57**	21.14**	207.33**	23.72**	10.95**	45.78**	36.31**	6.57**

**p<0.01.

### 3.5 Correlation analysis between relative seed germination and growth indices under combined salt-alkali stress

**Figure 5:**
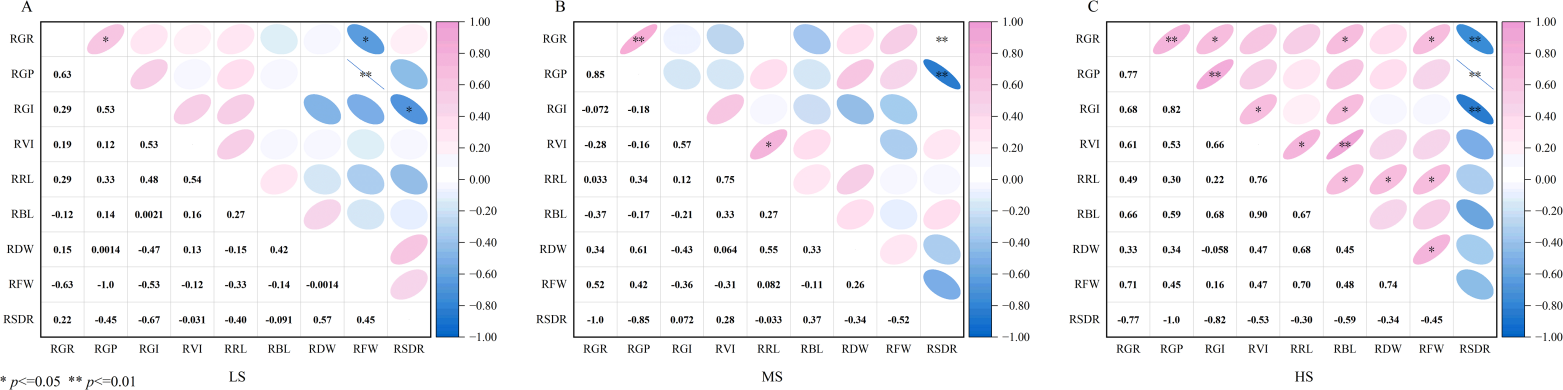
Correlation analysis of relative germination parameters in halophytes under low, moderate, and high combined salt-alkali stress. RGR, Relative germination rate; RGP, Relative germination potential; RGI, Relative germination index; RVI, Relative vigor index; RGL, Relative germ length; RRL, Relative radicle length; RFW, Relative fresh weight; RDW, Relative dry weight; RSDR, Relative salinity damage rate; (**A**) low combined salt-alkali stress; **(B)** MS, moderate combined salt-alkali stress; **(C)** HS, high combined salt-alkali stress. “*” indicated *p*<0.05, “**” indicated *p*<0.01.

Under combined salt-alkali stress, the correlations between the indicators showed differences (**Figure 5**). Low combined salt-alkali stress (**Figure 5A**): RGR and RGP (r = 0.63) showed a significant positive correlation (*p*<0.05), and RGI was significantly positive correlation with RVI and RRL (*p*<0.05). RRL was significantly positively correlated with RVI and RDW (*p*<0.05). RFW showed a very significantly negative correlation with RGR (r = -1.0) and RGP (r = -0.63) (*p*<0.01). RSDR was significantly negatively correlated with RGI (r = -0.67) (*p*<0.05). Moderate combined salt-alkali stress (**Figure 5B**): RGR and RGP showed a strong positive correlation (r = 0.85). RRL showed a significant positive correlation with RVI and RDW (*p*<0.05). RFW and RGR (r = 0.75) were significantly positively correlated (*p*<0.05). RSDR showed a very significant negative correlation with RFW (r = -1.0) and RGR (r = -0.85) (*p*<0.05). RVI was negatively correlated with RSDR (*p*<0.05). High combined salt-alkali stress (**Figure 5C**): RGR and RGP showed a very significant positive correlation (r = 0.77) (*p*<0.01). RVI and RGI showed a strong correlation (r = 0.90) (*p*<0.01). RSDR was significantly negatively correlated with all growth indicators, with the strongest negative correlation with RGR (r = -1.0) (*p*<0.01). The correlation coefficient between RFW and RSDR reached -0.82 (*p*<0.01).

### 3.6 Comprehensive evaluation using the membership function method on various relative indices during seed germination under combined salt-alkali stress

**Figure 6:**
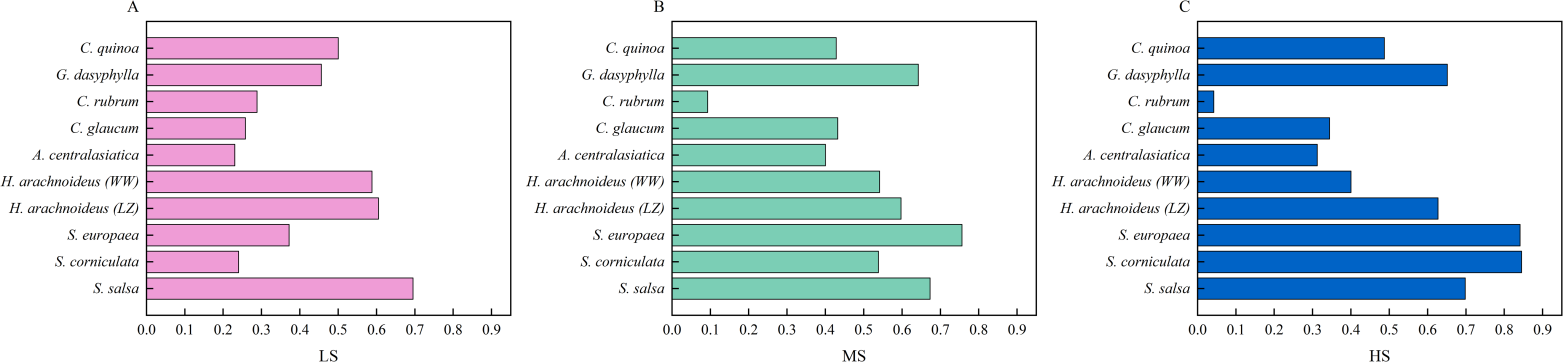
Comprehensive evaluation of halophytes at germination stage under low, moderate, and high combined saline-alkaline stress. **(A)** LS, low combined salt-alkali stress; **(B)** MS, moderate combined salt-alkali stress; **(C)** HS, high combined salt-alkali stress.

In this experiment, the fuzzy membership function method was used to comprehensively evaluate RGP, RGR, RGI, RVI, RLL, RGL, RFW, RDW, and RSDR of different halophytes. The results (**Figure 6**) showed that, the *D* value under low combined salt-alkali stress (**Figure 6A**) was 0.230~0.695, and the order of the *D* values was *S. salsa*>*H. arachnoideus* (LZ)>*H. arachnoideus* (WW)>*C. quinoa*>*G. dasyphylla*>*S. europaea*>*C. rubrum*>*C. glaucum*>*S. corniculata*>*A. centralasiatica*; the *D* value under moderate combined salt-alkali stress (**Figure 6B**) was 0.093~0.756, and the order of the *D* values was *S. europaea*>*S. salsa*>*G. dasyphylla*>*H. arachnoideus* (LZ)>*H. arachnoideus* (WW)>*S. corniculata*>*C. glaucum*>*C. quinoa*>*A. centralasiatica*>*C. rubrum*; under high combined salt-alkali stress, the *D* value was 0.042~0.845, and the order of the *D* values (**Figure 6C**) was *S. corniculata*>*S. europaea*>*S. salsa*>*G. dasyphylla*>*H. arachnoideus* (LZ)>*C. quinoa*>*H. arachnoideus* (WW)>*C. glaucum*>*A. centralasiatica*>*C. rubrum*.

## 4 Discussion

Under combined salt-alkali stress, halophytes seed germination strategies are categorized into “fast-track” and “slow-track” types (Petrovic et al., 2016). In this study, seeds of *S. corniculate*, *H. arachnoideus* (LZ), and *C. quinoa* complete germination within merely 1~3 days. This fast-track germination mechanism enables seeds to germinate rapidly and establish roots within a limited favorable window in regions with extremely scarce precipitation or extreme saline-alkali habitats, thereby ensuring survival (Debez et al., 2018). Conversely, species such as *S. salsa*, *S. europaea*, *H. arachnoideus* (WW), *C. rubrum*, *G. dasyphylla*, *A. centralasiatica*, and *C. glaucum* complete germination within 5~7 days. This slow-track germination strategy prevents all seeds from germinating simultaneously under stress and suffering collective damage, thereby enhancing the population’s survival probability. This strategy is similar to the low-risk germination strategy observed in the black seeds of *Suaeda aralocaspica* (Wang et al., 2012).

Combined salt-alkali stress on halophytic plants exhibits a typical “low promotion and high inhibition” phenomenon during seed germination and seedling growth (Wang et al., 2023a). Under low combined salt-alkali stress conditions, the fresh weight of all halophytes was higher than that of the control. The dry weight of *S. salsa*, *S. corniculata*, *H. arachnoideus* (LZ), *H. arachnoideus* (WW), *G. dasyphylla*, *C. quinoa* also reached their maximum dry weight under all salinity stress treatments. Indicating that low combined salt-alkali stress promotes the growth and biomass accumulation of most tested plants. This phenomenon may be related to the “trigger effect” of saline-alkali stress (Zamberlan, 2023; Zhai et al., 2025). *S. europaea, A. centralasiatica*, and *C. glaucum* exhibited optimal growth under moderate combined salt-alkali stress conditions, with fresh weights significantly higher than those under low and high treatments, 18.73%, 9.87%, and 13.42% higher than the LS treatment; 71.76%, 88.95%, and 127.46% higher than HS treatment, respectively. Indicating that these plants have a special adaptability to moderate saline-alkali environments. As intensity of combined salt-alkali stress increased, the inhibitory effect gradually became apparent. The fresh weight of all test plants under high combined salt-alkali stress conditions was lower than that of the control group. This growth inhibition may be due to ion toxicity caused by high concentrations of saline-alkali, which affects seed germination and seedling growth (Hu et al., 2022).

The most direct effects of combined salt-alkali stress on halophytes seed germination are decreases in RGP, RGR, RGI, and RVI (Tang et al., 2024). This study demonstrates that the relative germination indices of halophyte seeds are increasingly inhibited as the proportion of combined salt-alkali stress increases. In the initial germination stage, this inhibition may arise because the saline-alkaline solution reduces the water potential surrounding the seeds, thereby impeding water uptake (imbibition) (Li et al., 2022). Even if seeds ultimately complete imbibition, consequently suppressing germination metrics (Gul et al., 2013). It may be because that saline-alkali stress can significantly inhibit seed swelling and reduce α-amylase activity through osmotic stress, ion toxicity and oxidative damage, resulting in a decrease in germination potential and germination rate (Zhang et al., 2024). Furthermore, as the stress intensity (particularly salinity concentration) further escalates, the water potential gradient between the seed and its external environment significantly intensifies. This leads to dehydration of seed coat cells and potential structural damage to the cell walls, triggering solute leakage from the cells. Consequently, the supply of nutrients and energy required for germination becomes insufficient, exacerbating the inhibition of the germination process (Foster and Miklavcic, 2017). Due to differences halophytes species, the responses of different tissues and organs under salt stress and alkaline stress also vary (Vitali et al., 2021). In some plants, the inhibitory effect on the aboveground parts is more pronounced than on the underground parts (Li et al., 2021), while in others, the underground parts are more sensitive than the aboveground parts (Yang et al., 2022). During the germination period of halophytes in this study, a common phenomenon was observed, the inhibition effect of the same combined salt-alkali stress on RRL was significantly stronger than that on RGL. This may be attributed mainly to two factors: on the one hand, the radicle is the organ that breaks through the seed coat first and comes into direct contact with the stress environment during germination (Rajappa et al., 2024), and is in direct contact with saline solution; on the other hand, root cells are highly sensitive to stress factors, particularly under high pH conditions. It may be because that high pH and high salt stress inhibited radicle elongation by destroying root activity, causing osmotic imbalance, oxidative damage and ion toxicity (Katuwal et al., 2020). Ferreira et al. (2023) studied the pH is a limiting factor for seed germination, as well as for the initial radicle growth of *Zeyheria tuberculosa* seedlings. The results of this study support this view, as root length inhibition gradually increases with rising pH value.

Halophytes species, saline-alkali types, and their interactions jointly regulate germination and growth dynamics under saline-alkali stress, with stress intensity being a key regulatory factor (Ullah, 2024). Different plant groups exhibit differentiated adaptation strategies in different saline-alkali environments. Association analysis between seed germination and growth indicators under saline-alkali composite stress in this study revealed significant positive correlations: between RGP and RGR, and between RVI and RRL. These correlations demonstrate a synergistic effect between initial germination vigor and final germination efficiency. Furthermore, individuals with faster germination rates typically exhibit stronger physiological vitality and more developed root systems (Zhang et al., 2021). This suggests that, under the conditions of this study, early germination potential shows promise as an effective predictor for final seedling emergence rates in saline-alkali soils. Under combined salt-alkali stress, seeds adapt by rapidly germinating to shorten the salt-sensitive phase and accelerate entry into the autotrophic growth stage. This is synergistically coupled with prioritized root development to establish osmotic adjustment capacity (Ibrahim, 2016). This collective mechanism, rapid germination synergized with root prioritization, represents a core adaptive strategy for seeds confronting such adversity. Consequently, it provides clear target traits for breeding stress-tolerant varieties and optimizing cultivation practices. The RSDR was negatively correlated with RGI, RFW, and RGR, suggesting that salt action significantly reduces seed germination efficiency, inhibits plant physiological activity, and may temporarily slow biomass accumulation. RRL is positively correlated with RFW and RDW, indicating that plants with well-developed root systems can maintain higher biomass accumulation efficiency under salt-alkali stress conditions. This study also found significant differences in the pattern of correlations among indicators under different stress intensities: under low stress conditions (Liu et al., 2023), halophytes tend to adopt a rapid germination strategy, but this may reflect a resource allocation trade-off at the expense of biomass accumulation; under moderate stress conditions, the maintenance of high germination rates is essential for acclimatization, and this higher germination success is likely to be associated with enhanced water retention capacity to enhance acclimatization; and under high salinity stress conditions, salinity stress becomes the dominant limiting factor, exerting an overall inhibitory effect on plant growth and development.

The effects of low, moderate, and high combined salt-alkali stress on different halophytes are the combined result of multiple factors, and a single indicator cannot objectively and accurately reflect the tolerance of halophytes (Lyu et al., 2025). Therefore, this study used fuzzy membership function methods to comprehensively evaluate the salt tolerance of halophytic plants based on their relative germination and growth indicators in response to different combined salt-alkali stresses. In this study, *S. corniculata, S. europaea*, and *S. salsa* were found to be suitable for saline-alkali environments with a salt content of 0.73% and a pH of 9.63, demonstrating potential for cultivation and improvement in high saline-alkali soils. *S. europaea, S. salsa*, and *G. dasyphylla* are suitable for saline-alkali environments with a salt content of 0.55% and a pH of 8.90, and have moderate potential for cultivation and improvement in saline-alkali soils. *S. salsa* and *H. arachnoideus* (LZ)re suitable for saline-alkali environments with a salt content of 0.27% and a pH of 8.03, and have potential for cultivation and improvement in low saline-alkali soils. Compared with other halophytes, S. salsa showed stronger comprehensive salt tolerance stability. *C. rubrum and A. centralasiatica* showed weak salt tolerance stability and were more sensitive to stress intensification. *S. europaea* exhibited notably high germination rates (80%) under moderate combined salt-alkali stress, highlighting its advantageous performance, and an even higher rate of 84% under high combined salt-alkali stress. This pattern diverges from the typical “low-promotion, high-inhibition” trend. Concurrently, this species demonstrates characteristically low germination indices and prolonged, staggered germination. These traits, combined with its unique physiological adaptations, namely, a compartmentalized seed coat substructure (Lv et al., 2012) and a specialized multi-compartment vacuolar Na^+^ sequestration mechanism (Rosales-Nieblas et al., 2025), collectively constitute a distinctive high-salinity-alkalinity adaptation strategy. This integrated suite of adaptations enables it to maintain robust germination capacity across a broad spectrum of stress intensities, particularly excelling under elevated salinity-alkalinity conditions. *S. corniculata* has the highest *D* value under high combined salt-alkali stress, which is an ideal candidate species for further study of physiological and biochemical mechanism of plant saline-alkali tolerance.

In this study, with the increase of saline-alkali stress ratio, the saline-alkali tolerance of eu-halophytes was generally higher than that of pseudo-halophytes. This result is similar to the research of Liu et al. (2023) results on the salt tolerance ability of eu-halophytes such as *Suaeda physophora, Suaeda altissima* and *Kalidium foliatum* claw in germination stage is stronger than that of pseudo-halophytes such as *Limonium gmelinii, Limonium aureum* and *Limonium otolepis*. This divergence in saline-alkali tolerance likely stems from their distinct adaptation strategies during the germination stage. Pseudo-halophytes rely on specialized excretory structures (such as salt glands or salt bladders) to secrete and excrete excess salts. However, during the initial seed germination phase, these excretory organs are not yet differentiated, rendering them incapable of effectively removing absorbed saline ions from their tissues. Eu-halophytes, conversely, primarily employ a vacuolar compartmentalization strategy. Critically, even in the early germination stage, their embryonic tissues can actively transport and sequester absorbed ions like Na^+^ into vacuoles. This process significantly reduces cytoplasmic ion toxicity and utilizes the accumulated ions for osmotic adjustment to maintain cellular turgor pressure (Lu et al., 2021). It is precisely this vacuolar compartmentalization mechanism, effectively operational from the germination stage, which enables salt-accumulating plants to exhibit superior comprehensive tolerance compared to salt-secreting plants when facing escalating saline-alkali stress.

## 5 Conclusions

The study further explains the adaptation responses of ten halophyte plants to combined salt-alkali stress in relation to seed germination and seedling growth. Results: Most of the halophytes were significantly promoted by low combined salt-alkali stress (salt content 0.27%, pH 8.03, EC 3.62 mS cm^-1^) and had a “low-promoting” effect, among which 5 plants such as *S. salsa*, *S. corniculate*, and *H. arachnoideus* exhibited significant promotion effects on biomass accumulation. Under moderate combined salt-alkali stress (salt content 0.55%, pH 8.90, EC 6.38 mS cm^-1^), *S. europaea* and *A. centralasiatica* maintained acclimatization by delaying germination or optimizing root development; heavy combined salt-alkali stress (salt content 0.73%, pH 9.63, EC 9.54 mS cm^-1^) generally inhibited growth, eu-halophytes (*S. europaea* and *S. corniculata*) outperformed pseudo-halophytes (*C. glaucum* and *C. rubrum*). The comprehensive evaluation indicates that *S. salsa* possesses broad-spectrum tolerance to saline-alkali stress, while *S. corniculata* and *S. europaea* exhibit remarkable tolerance to high and moderate combined salt-alkali stress, respectively. Seed germination strategies (“fast-track” and “slow-track” types) are significantly correlated with salt-alkali tolerance, and the extent of root development is positively correlated with biomass accumulation. This study provides a theoretical basis for species selection in the biological remediation of saline-alkali land. 

## Data Availability

The original contributions presented in the study are included in the article/supplementary material. Further inquiries can be directed to the corresponding author.
